# Without Medicine

**Published:** 1879-04

**Authors:** 


### WITHOUT MEDICINE,

It has been seen how invariably the derangement of pulse and breathing go together, showing that the cause is one, and the locality the same, the Lungs. As the heart is always pumping its blood into the lungs, to present it to the action of the air, in order to render it fit for vital purposes, the faster the pumps work, the faster must the lungs work. But what makes the heart work faster? The blood in it is more impure than natural, that is, more thick, it does not flow with ease, it is sluggish, each motion of the heart does not get rid of its proper quantity, and it must work faster or drown; as the refractory poor in the workhouse, who are unwilling to work, and are placed in a large tank or tub, into which water is pumped, and they have the alternative of pumping with another pump, or drowning. This thickened nature of the blood makes itself felt in the lungs, in the same way as in the heart, with the additional effect of the formation of tubercles, and these taking up more room in the lungs, leave less room for the requisite amount of air, the person must breathe faster and consequently shorter, the result being to aggravate the difficulty. Thus it is that consumption does not get well of itself, like many other diseases, any more than a fire will go out of itself, until it has left the building in ashes, unless for the want of one of two things—a want of burning material or an artifical barrier. But in consumption, there is material, as long as there is a body; and how it is destroyed, until nothing is left but skin and bone, we need no information! The only remedy then, is the artificial barrier. *What is it?*

But before replication is made to that inquiry, it is practically useful to go another step more remote in our inquiries in the way of a reminder. What makes the blood thus preternaturally impure in the heart, so as to lay the foundation for such vast destruction? This is answered in preceding pages, beginning at p. 168, vol. 2, where it is shown that the fundamental origin of impure, consumption-originating blood is, imperfect nutrition and the habitual breathings of a still atmosphere in-doors. And let it be painted before the mind's eye in living light, that either of these causes can alone certainly originate consumption, however wholly and completely the other may be absent. That all our care as to our food will not save us from consumption, if we habitually breathe a confined air. Nor will an active out door life save us from consumption or other fatal disease, if we live upon improper food, or habitually eat more of the best food in the world, than the digestive functions can turn into pure nutrient blood material.

Here then, we are brought square up to the important inquiry, the prevention, the permanent arrest, or lasting cure of consumption. It is found

"In the Food we eat—In the Air we Breathe."

A perfect digestion of wholesome nutritious food, and a habitual breathing of out door air, under circumstances of proper bodily activity, is competent to cure consumption, from its first beginnings to its last stages, that is, the stage of actual decay of the lungs.

But as very few, in the latter stages, possess the energy requisite to secure the amount of out-door activity, necessary to the proper digestion of substantial food, we must go back to a point where we can secure the intelligence of the parent, acting authoritatively over the child. There must be Light and Force. There is power in concentration. And it is of interest to inquire, to which of the two causes of blood impurity, is the origin of consumption most attributable? Then, by directing most of our energies to that one principal cause, we may act more efficiently. A stream of water puts out a fire, if played on one spot, but may be wholly unavailing, if thrown over the whole building.

The consummating act of Creative Power was to make man. The consummating act of Infinite Beneficence, is his preservation. We evidently were made to people the globe; wherever we live, we must subsist. Thus we find that the stomach makes out of all things, one thing, a fluid mass, which does not materially vary in color, consistency or nature, whatever we may eat. So that in a modified sense, we can, in health, derive nutriment from almost any thing we can swallow, from the lion to the worm; from the eagle to the insect; from the tree bud to its root, whether leaf or fruit, or bark or wood. Hence then, we come to an important practical fact: In consumption a man may eat almost any thing, if judicious as to quanity. Thus it is, that uniformly, we have, in our own practice, *as a general rule*, given the broad direction: Eat what you

LIKE, and which is not followed by any uncomfortable feeling within an hour or two afterwards.

It is a truth which should be kept sight of in all human maladies, that great Nature is our safest and wisest Teacher, and with an almost unerring instinct creates in us a desire for that kind of food which contains in it those elements which the body most needs at the time. An instructive illustration, occurring within a few years, may not be out of place at this point, as serving to impress an important truth on the mind:

A girl fell down a flight of stairs, receiving an injury from which it was thought she would not recover. But with the exception of hearing and sight, she did recover. For some weeks her appetite called for nothing but raisins and candy, then for several months nothing but apples were eaten. At a later period, she commenced eating maple buds, since which time she has nearly regained her former health, and at the end of three years, her sight and hearing were restored.

We knew a child, twelve months old, abandoned to die by several of the most skilful physicians of New York, from teething and attendant summer complaint. As a last resort, it was sent to the sea shore in a two hours journey; on arriving there in a cold raw afternoon of August, the only attainable thing that seemed at all suitable, was a bowl of boiled milk, which she took ravenously, and would take nothing else for a week, improving from the first hour, and at end of a year is among the heartiest and most rugged of children. And to make the prescription more impressive, having nature still on our side, we say to those under our care:

Let no man's appetite be a guide for your stomach; but only eat what you crave, even if it be a piece of pound cake or sole leather; eat it in great moderation first, so as to be on the safe side, and gradually increase the quantity. On the other hand, never swallow an atom which you do not crave, for nothing nor nobody. A pig would not so violate nature. It should strike us as one of the most reasonable of inferences, that the stomach would most easily digest that which it most eagerly craved. There are morbid and unnatural cravings, but these are exceptions. We are speaking as to general rules, here and elsewhere in this volume, and it will help the reader to a more truthful appreciation of the principles advocated in these pages, if this distinction is kept clearly in view.

If then in the two great points of digestion and out door activities, the former may be, to a considerable extent lost sight of, as being, under a wise arrangement of providence, able to take care of itself, we naturally throw our whole attention to the other and only one great remedial means in consumptive disease, which is—

### OUT DOOR ACTIVITIES.

Any train of argument may look beautifully conclusive until a missing or unbelonging link is discovered; the removal of the latter or the replacement of the former, makes sad havoc sometimes, of splendid theories. But when facts coincide with theories in the management of consumption, there is a triumph for science well worthy of being recorded. And we are led to the inquiry:

### DO OUT DOOR ACTIVITIES CURE CONSUMPTION?

If in answering this important question, we gave cases coming under our own management, they might be questioned as to their authenticity, by reason of our personal interest in the same. So we will first give a history or two from undoubted medical authority.

EDENTOWN, N. C., *February*, 1830.

DR. PHYSIC, *Philadelphia*—DEAR SIR:

In the month of April, 1812, after having been extremely reduced by an attack of bilious fever, I was seized with a cough, which continued, with great obstinacy and severity, until the month of November, when decided symptoms of Phthisis (consumption) began to make their appearance. I had every evening an exacerbation (recurrence) of fever, preceded by chilliness, and succeeded by copious perspiration. My cough began to be less painful, but was attended with an expectoration of mucus, mixed with pus, (yellow matter.) Before this complaint came on me, I had accepted a surgeon's commission in the army, and was stationed at Tarborough, about seventy-five miles from this place. In the month of December the part of the regiment which had been recruited, then having been ordered to Salisbury, it became my duty to repair to that place.

"Accordingly, about the middle of the month, in the situation I have described, I set out on my journey.

"In two days I reached Raleigh, without having experienced any material change in the symptoms of my complaint. During my stay in Raleigh, the disease increased every day, so that I was obliged to remain there nearly a week, at the expiration of which time I had almost determined to retrace my steps, return home, and take my station among the forlorn and despairing victims of this unrelenting malady.

"But reflecting deeply on my situation, and recollecting that scarce a patient in a thousand had been known to recover from the disease after having been confined to bed by it, I was resolved to resume my journey, and to reach the place of destination or perish on the road. It will be impossible for me ever to forget the effort I had to make in pursuing this resolution. On a cold and blustering morning about the 20th of December, weak and emaciated, having been literally drenched in perspiration the night before, I ascended my gig and proceeded on my journey. The first part of my ride, this day, was excessively irksome and fatiguing. Every hovel and hamlet on the road seemed to invite me to rest, and to dissuade me from the prosecution of my undertaking. Often and anxiously did I wish that my disease had been of such a nature as to allow me to indulge in the inclination I felt, to desist from motion. But I continued my ride for three hours, when I found it necessary to stop for a little refreshment. While dinner was preparing, I lay down on a bed to rest. It was, perhaps, an imprudent act. Never was a bed so sweet to the wayworn and exhausted traveller, as was this to me. I lay on it for an hour, wrapped, as it were, in elysium. When summoned to dinner, though sleep was fast stealing on me, and inviting me to be still, I arose and attended, and after having made a very moderate meal of very common country food, I resumed my ride, and at night, about half past six o'clock, arrived at Hillsborough, which is distant about 36 miles from Raleigh. The inn to which I had been recommended was unusually crowded, and I had to accept of a room that was out of repair, the window-sashes rattling in their casements, and the wind passing through the sashes in several places. In such a chamber, at such a season, and in the situation already described, was I quartered for the night. To my surprise, however, I had a better night's rest than I had had for several weeks, and less perspiration, and coughed less than I had for a month before

"In the morning, considerably refreshed, I proceeded on my journey, and travelled in a foggy misty atmosphere full 40 miles; the next day about 35, and on the 4th day about 12 o'clock, I arrived at Salisbury. On my arrival, I heard it mentioned as a matter of astonishment, that a man in my situation should think of travelling in the cold and inclement season of winter; much more astonishing that I should venture to approach the mountains at such a period. But I had taken my resolution, and was determined never to relinquish it while I had power to walk or ride. The regiment to which I was attached, was encamped about four miles from the town of Salisbury. To this place I tasked myself to ride twice every day, a duty I regularly performed in the coldest weather until I left the service.

"Early in January the officer in command received orders to repair with his regiment to Canada. While preparations were making for that purpose, believing that such a climate would be too severe for me, and that I must of course soon cease to be useful to the Government, I addressed a letter to the Secretary of War, soliciting permission to retire from the army. This request was promptly and kindly granted to me. In February, 1813, I commenced the practice of my profession again in this place, and continued to attend to the most laborious duties of it at all times of the day and night, in rain, hail, snow, storms, and sunshine, whenever I was called on, for eighteen months.

"At the end of that time, I had lost my hectic fever, night-sweats, purulent expectoration, and my cough had nearly left me; my chest had recovered its capacity of free and easy expansion, and the ulcers in my lungs had entirely healed. Many who read the foregoing statement, will no doubt be curious to know what medical means were used as auxiliaries in the cure of this very alarming state of disease. It would not be in my power to satisfy curiosity on this point were it a matter of any importance, which I conceive is not the case, *the complaint having been cured by hardy, invigorating exercise, continued without interruption in every variety of temperature and weather.*

"That palliatives of different kinds were resorted to at various periods, must at once be supposed, but I do not consider it a matter of consequence to name them, as they were such as would readily suggest themselves to physicians of every grade of skill or intellect, and never produced more than a temporary alleviation of symptoms. Perhaps it may be material to state, I never used opium in any form whatever, and that I never incautiously wasted the resources of my constitution by depletory, or debilitating means. When symptoms of high arterial excitement occurred, which would sometimes be the case, it was my practice to abstain from strong, high-seasoned food, from all fermented and spirituous liquors, and from active exercise until they subsided. By this negative mode of management I generally succeeded in removing inflammation without materially impairing the energies of my system; and on the increase of the purulent discharge, subsequent to such inflammatory appearances, I betook myself again to my exercise, and ate and drank everything I wanted. I always found that the inconvenience produced by a full meal, yielded very soon to horse exercise, and that I generally coughed less while riding than at any other time. The hectic paroxysm was generally interrupted, and sometimes cut short by a hard ride, and often, very often, during the existence of my disease, have I checked the exhausting flood of perspiration, and renewed my strength and spirits, by turning out of bed at midnight and riding a dozen miles or more; many a time, too, have I left my bed in the early part of the night, wayworn with coughing, restlessness and sweating, for the purpose of visiting a patient, and after having rode an hour or two, returned home and slept quietly and refreshingly for the remainder of the night.

"Another thing which I remarked in the course of my experience in the disease was, that some of the most profitable rides I ever took were made in the coldest and most inclement weather, (air dense and plenty of oxygen for assimilation,) and that scarcely in any situation did I return from a long and toilsome ride, without receiving a sensible amendment in all my pulmonary complaints. In short, sir, were I asked to state in a few words the remedy which rescued me, I should say it was a life of hardy exercise and of unremitting toil, activity, and exposure. With pectorial medicines, or those articles or compositions denominated expectorants, I seldom meddled in my own case; without opium, which from a constitutional peculiarity, I have not been able to take for many years, I found them too debilitating; and with it, had I been able to use the article, I should not have been disposed to take them, lest their effect in disposing to rest and inactivity might have operated against the course I had prescribed for myself, and from which I expected relief.

"It remains for me to mention another agent which I think excited a very curative influence upon my disease, and that is singing. In first using this remedy it was my custom to sing in a low tone, and not long at a time, so as not to occasion much pulmonary effort. But by degrees I became able to sing in the most elevated tones, and for hours together, allowing myself only such intervals of rest as the lungs required to obviate injurious fatigue. So long and so frequently did I repeat this act in the course of my disease, that the exercise of singing became so strongly associated, that as soon as I mounted my horse or ascended my chaise, I found myself humming a tune, and often in my lonely rides through the country, at late and unseasonable hours of the night, have I made the woods vocal with the most exhilarating music. Singing seemed always to have the effect of clearing the bronchial passages, of opening the chest, and of giving a greater capacity of motion and expansion to the lungs. [The Doctor was killed by accident, in 1850.] "Yours, etc., JAMES NORCOM."

Dr. Norcrom mentions a case as having occurred in 1810, which in 1830, twenty years later, was wholly free from any disease of the lungs. All this patient did, was to ride ten miles a day, gradually increasing to twenty miles a day, and by a continuance of exercise, was eventually restored to perfect health. All the medicine this man took was tincture of digitalis; but as it is now generally acceded that this remedy is worthless in consumption, the cure must be attributed to the exercise, just as the following case as given by Dr. Stokes, whom we have personally known at his own home in Dublin; and whom we found to be, as is universally accorded by the profession, among the very foremost of living medical minds. The case was first reported in one of the British medical periodicals in 1854, and republished here in April of the succeeding year.

"Some years ago I saw a gentleman who came to town laboring under all the symptoms of well-marked phthisis. The disease had been of several months' standing, and the patient *was*

*a perfect picture* of consumption. He had a rapid pulse, hectic, sweating, purulent expectoration, and the usual *physical signs* of tubercular deposit, and of a cavity under the right clavicle. I may also state, that the history of the disease was in accordance, in all particulars, with this opinion. I saw this patient in consultation with a gentleman of the highest station in the profession, and we both agreed there was nothing to be done. This opinion was communicated to the patient's friends, and he was advised to return to the country. In about eighteen months afterwards, a tall and healthy-looking man, weighing at least twelve stone, entered my study with a very comical expression of countenance: "You don't know me, Doctor," he said. I apologised, pleading an inaptitude that belongs to me for recollecting faces. "I am," he said, "the person whom you and Dr. —— sent home to die last year. I am quite well, and I thought I would come and show myself to you." I examined him with great interest, and found every sign of disease had disappeared, except that there was a slight flattening under the clavicle.

"'Tell me,' said I, 'what have you been doing?' 'Oh!' he replied, 'I found out from the mistress what your opinion was, and I thought as I was to die I might as well enjoy myself while I lasted, and so I just went back to my old ways.' 'What was your old system of living?' said I. 'Nothing particular,' he said, 'I just took what was going.' 'Did you take wine?' 'Not a drop,' he replied, 'but I had my glass of punch as usual.' 'Did you ever take more than one tumbler?' 'Indeed I often did.' 'How many: three or four?' 'Ay, and more than that: I seldom went to bed under seven!' 'What was your exercise?' 'Shooting,' he said, 'every day that I could get out.' 'And what kind of shooting?' 'Oh! I would not give a farthing for any kind of shooting but the one.' 'What is that?' 'Duck shooting.' 'But you must have often wetted your feet.' 'I was not very particular about the feet,' says he, 'for I had to stand up to my hips in the Shannon for four or five hours of a winter's day following the birds.' So, gentlemen, this patient spent his day standing in the river, and went to bed after drinking seven tumblers of punch every night; and if ever a man had recovered from phthisis he had done so when I saw him on that occasion.

